# What do students really gain from physical education? The QPE4PL*impact* as a situational measure of physical literacy in high school

**DOI:** 10.3389/fpsyg.2026.1876341

**Published:** 2026-07-20

**Authors:** Matthieu Stioui, Lisa Lefèvre, Cédric Roure, Oguzhan Aksoy, Christophe Schnitzler

**Affiliations:** 1 Faculty of Sport Sciences, Strasbourg, France; 2 E3S (EA1342) “Sport and Social Sciences”, Strasbourg, France; 3 HEP Vaud, Lausanne, Switzerland

**Keywords:** high school, physical education, physical literacy, questionnaire, validation

## Abstract

**Introduction:**

Existing physical literacy (PL) assessment tools primarily evaluate enduring traits and provide limited insight into students’ immediate experiences during physical education (PE) lessons. This study aimed to develop and validate the QPE4PL*impact*, a questionnaire designed to assess the situational impact of PE lessons on high school students’ PL. Grounded in a monist and phenomenological perspective, PL was conceptualized as an embodied and contextual construct encompassing physical, psychological-affective, cognitive, and social domains.

**Methods:**

Questionnaire development followed a three-phase process involving (1) domain identification based on existing theoretical frameworks and expert consensus, (2) item generation and refinement via expert feedback and cognitive interviews, and (3) psychometric evaluation with exploratory and confirmatory factor analyses.

**Results:**

The analyses supported a four-factor, 19-item structure with good reliability and satisfactory evidence of construct validity.

**Discussion:**

The QPE4PLimpact provides a theoretically grounded and pedagogically relevant instrument for assessing the immediate, context-dependent expression of PL in PE lessons. It offers new opportunities for evaluating educational practices and supports future cross-cultural and longitudinal research.

## Introduction

Physical literacy (PL) is increasingly viewed as a key construct for understanding lifelong engagement in physical activity and well-being across the lifespan. Defined by International Physical Literacy Association (2017) as “the motivation, confidence, physical competence, knowledge and understanding to value and take responsibility for engagement in physical activities for life,” PL encapsulates a comprehensive view of human movement that interconnects body, mind, and context. It foregrounds the idea that physical activity is not a purely motor endeavor but an integrative expression of physical, psychological-affective, cognitive, and social capacities developed through lived experience.

Conceptually, PL is recognized as a multidimensional construct encompassing four interrelated domains: the physical (competence and movement skills), psychological-affective (motivation, confidence, self-determination), cognitive (understanding, knowledge, awareness of strategies), and social (interaction, cooperation, responsibility) domains. These domains operate in dynamic interplay, collectively shaping an individual’s ability and willingness to engage meaningfully in movement across varying contexts.

The philosophical foundations of PL lie in monism, existentialism, and phenomenology (Merleau-Ponty, 1945; van Manen, 1990; Whitehead, 2001), which reject Cartesian dualism by positioning movement as an embodied, situated, and meaningful experience. Within this perspective, learning and personal development are understood as processes emerging through lived interactions between individuals and their environments. Consequently, PL is increasingly conceived not as a fixed trait but as an evolving journey shaped by experiences accumulated over time (Green et al., 2018).

These theoretical foundations have important implications for assessment. If PL is multidimensional, dynamic, and context-dependent, its measurement should not be restricted to static or decontextualized evaluations but should instead acknowledge the situational experiences through which PL develops. Accordingly, the assessment of PL has become one of the most active areas of research within the field over the past 25 years (Carl et al., 2023). Several systematic reviews have documented a rapid expansion in the number of available instruments (Barnett et al., 2023; Edwards et al., 2018; Jean de Dieu and Zhou, 2021).

Existing PL instruments can broadly be grouped into three categories. A first category includes self-report questionnaires designed to assess perceived PL, such as the Perceived Physical Literacy Instrument (PPLI) (Sum et al., 2018) and the Physical Literacy in Children Questionnaire (PL-C Quest) (Barnett et al., 2022). These instruments offer practical and accessible ways of capturing individuals’ perceptions of their PL but rely exclusively on self-reported data. A second category combines subjective perceptions with objective assessments of physical competence. Examples include the Canadian Assessment of Physical Literacy (CAPL-2) (Longmuir et al., 2018), the PLAY Tools (Caldwell et al., 2020), and the Emerging Adult Physical Literacy Assessment (ELIP) (Gandrieau et al., 2023). These instruments provide a more comprehensive evaluation of PL by integrating multiple domains and assessment methods, but often require substantial time, resources, and trained personnel. A third and more limited category includes instruments developed specifically for physical education (PE) settings, such as the Portuguese Physical Literacy Assessment (PPLA) (Mota et al., 2021), which was designed for secondary school students and demonstrates strong validity evidence within educational contexts.

Despite these advances, several limitations remain. Most existing instruments assess PL as a relatively stable or global construct rather than capturing how it is experienced within a specific learning situation. In addition, few tools have been specifically developed for older adolescents, and even fewer focus on the immediate educational context of PE lessons. Consequently, current assessment approaches provide limited insight into how students perceive the physical, psychological-affective, cognitive, and social outcomes of a particular PE experience. This limitation appears especially important given the philosophical foundations of PL, which emphasize embodiment, contextuality, and the role of lived experiences in shaping development over time.

Despite the growing number of PL assessment tools, a validated instrument specifically designed to capture the immediate impact of PE lessons on high school students’ PL remains lacking. This gap is particularly important given that adolescence represents a critical developmental period during which lifelong physical activity habits are shaped, while participation in physical activity often declines (Van Sluijs et al., 2021). Moreover, the predominance of global or trait-oriented assessments appears only partially aligned with the philosophical foundations of PL, which emphasize lived experience, contextuality, and the dynamic interaction between individuals and their environments. Consequently, there is a need for measurement approaches capable of capturing how students experience and perceive the physical, psychological-affective, cognitive, and social outcomes of a specific PE lesson.

Therefore, the purpose of this study was to develop and validate the QPE4PL*impact* questionnaire, a situational measure designed to assess the immediate impact of PE lessons on high school students’ PL. Grounded in a multidimensional conception of PL, the instrument aims to capture students’ perceptions across the physical, psychological-affective, cognitive, and social domains within the specific context of a PE lesson. To achieve this objective, the study followed a rigorous instrument development process involving subdomain identification, item generation and refinement, exploratory factor analysis (EFA), and confirmatory factor analysis (CFA).

## Methods

### Overview of the study

The development and validation of the QPE4PL*impact* questionnaire followed a three-phase process. Phase 1 focused on the identification of the PL domains and subdomains to be assessed, based on existing theoretical frameworks and expert consensus. Phase 2 involved item generation and refinement through expert review and cognitive interviews with high school students. Phase 3 consisted of the psychometric evaluation of the questionnaire using EFA and CFA conducted on two independent subsamples.

### Phase 1: Domains and subdomains identification

The identification of relevant domains and subdomains of PL to be included in the QPE4PL*impact* questionnaire was grounded in the international expert consensus established by Gandrieau et al. (2023) through a rigorous Delphi process. This framework, endorsed by a multidisciplinary panel of PL specialists, encompassed physical, psychological-affective, cognitive, and social domains, each divided into operationalizable subdomains.

In the present study, an additional critical screening phase was conducted to ensure suitability for situational assessment in the context of a physical education lesson. Two inclusion criteria guided this refinement: (i) direct observability or immediate self-report validity immediately following a PE session, and (ii) construct relevance to the four-dimensional, monist conception of PL underpinning this study. Subdomains that required longitudinal observation, objective laboratory testing, or context-free reflection (e.g., benefits and risks, motor creativity) were excluded to preserve both ecological validity and feasibility. This careful selection enabled the development of a targeted yet theoretically aligned construct map, serving as the basis for item generation.

Application of the two inclusion criteria led to the retention of a parsimonious yet comprehensive set of subdomains within the four PL domains (Table 1). All psychological-affective subdomains identified by Gandrieau et al. (2023)—motivation, self-esteem, emotional competence, confidence, and enjoyment—were preserved, reflecting their central role in capturing immediate affective change post-lesson.

**Table 1:** Domains and subdomains identified by Gandrieau et al. and those retained for the development of the QPE4PL*impact* questionnaire.

Domain	Subdomain	Retained/Discarded
Psychological-Affective	Motivation	Retained
	Self-Esteem	Retained
	Emotional competence	Retained
	Confidence	Retained
	Enjoyment	Retained
Cognitive	Benefits and risks	Discarded
	Recommendations	Discarded
Physical	Objective tests	Discarded
	Movement competencies	Retained
	Perceived Aquatic competencies	Discarded
	Bicycle competencies	Discarded
	Motor creativity	Discarded
Social	Social acceptance	Retained
	Relational skills	Retained
	Self-management	Retained
	Social awareness	Retained
	Responsible decision-making	Retained

Within the cognitive domain, subdomains addressing generalized knowledge beyond the lesson context (e.g., benefits and risks, recommendations) were eliminated, as they lacked situational specificity. In line with this methodological decision, the questionnaire was deliberately oriented towards the assessment of cognitive processes that are enacted within the immediate context of the lesson, rather than general declarative knowledge. Accordingly, new items were designed to capture constructs such as decision-making, tactical awareness, and the understanding of game strategies—elements recognized as central to effective, in-situ engagement in physical activity.

This approach draws theoretical support from complementary models such as the Tactical Games Model (Oslin et al., 1998), which foregrounds game intelligence and context-dependent decision-making as core to the demonstration of competence in physical education settings. Furthermore, recent literature emphasizes that many existing PL assessment tools tend to overlook these dynamic cognitive aspects. For example, Shearer et al. (2021) highlight how the evaluation of PL often neglects crucial subdomains such as tactical knowledge, understanding of rules, and game strategies, despite their recognized importance for holistic development and meaningful participation.

By foregrounding these contextually anchored cognitive constructs, the present study addresses an identified gap and strengthens the ecological validity and educational relevance of situational PL assessment. The exclusion of certain subdomains from Gandrieau et al.’s original Delphi framework aimed to capture the in-the-moment cognitive processes essential to PL in educational settings.

In the physical domain, only movement competencies were deemed directly assessable through self-perception in an immediate post-activity survey; subdomains requiring objective verification, such as aquatic competencies or bicycle competencies, were excluded after agreement with a senior researcher acting as research director. All five social subdomains were retained, underlining their contextual relevance in PE settings where peer interaction is a core component. This selection created a streamlined construct map optimized for situational measurement without sacrificing theoretical breadth.

### Phase 2: Item generation and refinement

#### Item generation

A series of questionnaire items was developed to address each of the eleven retained subdomains. All items were collaboratively developed by two experts in the field of PL (a doctoral researcher and a full professor). The initial objective was to generate a minimum of four items per domain, drawing upon the available subdomains—a threshold commonly recommended to ensure the stability of reliability estimates and to enable robust evaluation of measurement models (Bollen, 1989). This requirement occasionally necessitated the inclusion of multiple items within the same subdomain. For example, the physical domain contained only one retained subdomain—physical competencies—which was represented by four items. Additional items were also developed in specific cases: (i) within the cognitive domain, where no exploitable subdomain remained after the refinement process. Because none of the cognitive subdomains identified in the Delphi consensus were considered suitable for a situational post-lesson assessment, they were not retained as formal subdomains. Nevertheless, the cognitive domain itself was preserved, and items were developed directly from theoretical constructs commonly associated with cognitive aspects of PL (e.g., decision-making, tactical awareness, and understanding of game strategies). These constructs therefore served as item-writing guides rather than as formal subdomains; (ii) within the social domain, to capture subdomains deemed contextually relevant; and (iii) within two subdomains of the psychological-affective domain (motivation and self-esteem), in order to create items differing according to the temporal framing of the question.

All items were formatted using a uniform 5-point unipolar response scale to maximize reliability and validity (DeCastellarnau, 2018; Furr, 2011). This format measured the intensity of agreement along a single continuum, with fully verbalized response categories ranging from “Strongly disagree” to “Strongly agree.” Such a design minimizes ambiguity, enhances respondent comprehension, and is recognized for improving the precision and interpretability of self-report instruments. To anchor responses in the situational context, each item began with the stem *“During today’s PE lesson…,”* encouraging participants to directly relate their answers to their immediate experience. The initial version of the questionnaire consisted of 27 items distributed across the four domains: 4 physical, 8 psychological-affective, 10 social, and 5 cognitive items.

#### Cognitive interviews

The cognitive interviewing phase aimed to verify item interpretability, cultural appropriateness, and context alignment. A randomly selected, sex-balanced sample of high-school students (15–18 years old) from diverse socio-educational settings participated in the study. Semi-structured interviews were conducted by a doctoral researcher within 15 min after a PE lesson in a classroom to exploit the immediacy of the experiential recall.

Using a verbal probing technique, students were invited to articulate their understanding of each item, paraphrase its meaning, and describe how they arrived at their chosen responses. Feedback was systematically coded to flag ambiguities, unintended connotations, and item redundancy (Beatty and Willis, 2007; Willis, 2004). This process generated insights into how situational factors (e.g., type of activity, group dynamics, teacher feedback) influenced response patterns, information later integrated into the refinement of the questionnaire.

A total of *n* = 16 students participated in cognitive interviews, each lasting approximately 20 min at most, producing both anticipated and unexpected findings. Most items were reported as easily understandable, with language appropriately calibrated for the target age group.

However, certain formulations**—**particularly those related to self-management in the social domain**—**were flagged for potential ambiguity, as students tended to interpret them either in terms of task organization or emotional self-regulation. For example, the initial item “we learned to manage ourselves autonomously” was perceived by some students as referring to organizing the activity or managing the group, rather than reflecting individual autonomy. To improve clarity and align the wording with the intended construct, the item was reformulated as “I feel that I have learned to act autonomously.”

Interview data prompted the simplification of complex syntactic structures in two items and the replacement of abstract terminology with concrete, lesson-specific references. Moreover, several students spontaneously commented on how group atmosphere and teacher feedback influenced their responses, reinforcing the situational validity of the tool. Incorporation of these refinements led to the final pilot version subjected to large-scale psychometric testing.

### Phase 3: Psychometric evaluation

#### Participants

The study population comprised students from four distinct high schools located in the South of France. A total of 493 students participated (274 girls, 219 boys), ranging from 10th to 12th grade (approximately 15–18 years old), completed the questionnaire immediately after a PE lesson. To enable robust psychometric testing, the sample was randomly split into two independent subsamples. Approximately 67% of participants (*n* = 328) were allocated to the exploratory sample and used for EFA, whereas the remaining participants (*n* = 165) constituted the confirmatory sample used for CFA. This cross-validation approach enhanced the generalizability and stability of the factorial structure.

#### Procedure

Data were collected in four high schools located in the South of France. Students completed the QPE4PL*impact* questionnaire immediately after a PE lesson to capture their situational perceptions of PL in the context of the lesson. The instrument was administered across a variety of curricular PE activities, encompassing both individual and team-based activities with differing physical, technical, and tactical demands. As the primary objective of the study was the validation of a situational PL instrument rather than the comparison of activity types, responses were analyzed collectively across activities. Participation was voluntary, and all procedures complied with ethical standards for research involving human participants. Written informed consent was obtained from all participating minors, with additional parental authorization secured prior to data collection. Institutional approval was granted by both the school principals and the Rectorat of the Académie d’Aix-Marseille. The study was conducted in accordance with the ethical principles of the Declaration of Helsinki.

#### Data analysis

All statistical analyses were performed using R (RStudio, version 2023.06.1). Prior to analysis, the dataset was examined for missing values. Missing responses represented 0.64% of the dataset and were imputed using the *imputePCA* function from the *missMDA* package in R. This approach was selected to preserve the multivariate structure of the data while avoiding information loss associated with listwise deletion.

Given the ordinal nature of the items**—**rated on a 5-point Likert scale**—**the factor analyses were conducted on a polychoric correlation matrix. Unlike the Pearson correlation coefficient, which assumes continuous and interval-level data, polychoric correlations estimate the relationships between latent continuous variables underlying the observed ordinal responses, thus providing more accurate association estimates for psychometric purposes.

Two preliminary statistical tests were conducted to confirm the suitability of the data for factor analysis. First, Bartlett’s Test of Sphericity was applied to evaluate whether the correlation matrix significantly deviated from an identity matrix, with *p* < 0.05 indicating patterned relationships suitable for factor extraction. Second, the Kaiser-Meyer-Olkin (KMO) measure of sampling adequacy quantified the proportion of variance potentially attributable to common factors. KMO values above 0.80 were considered “good,” and values exceeding 0.90 “marvelous,” supporting the appropriateness of factor analytic techniques.

EFA was conducted on the exploratory subsample using a polychoric correlation matrix and direct oblimin rotation. Factor retention was guided by the Kaiser criterion (eigenvalues > 1) and visual inspection of the scree plot. Items with factor loadings below 0.40 or substantial cross-loadings were iteratively removed.

The CFA was conducted on the confirmatory subsample (*n* = 165, ≈33%) to cross-validate the factor structure identified in the EFA. Models were specified using the *lavaan* package in R and estimated via the Weighted Least Squares Mean and Variance adjusted (WLSMV) estimator, chosen for its robustness to non-normality in ordinal data. Model fit was evaluated using multiple indices and established benchmarks: Comparative Fit Index (CFI > 0.85), Tucker-Lewis Index (TLI > 0.85), Root Mean Square Error of Approximation (RMSEA < 0.08), and Standardized Root Mean Square Residual (SRMR < 0.08).

To examine higher-order construct validity, a second-order CFA was conducted, testing the hypothesis that all first-order factors reflected an overarching PL construct. The model, estimated on the confirmatory subsample using WLSMV, was assessed using the same fit criteria as the first-order CFA. Strong model fit in this hierarchical structure would support the presence of a unifying latent dimension underlying the specific PL domains measured by the QPE4PL*impact*.

## Results

### Preliminary analyses

Preliminary diagnostics confirmed the suitability of the dataset for factor analysis. Bartlett’s Test of Sphericity was statistically significant (*p* < 0.05), indicating that the correlation matrix was not an identity matrix and that patterned relationships existed among variables. The KMO measure of sampling adequacy produced an overall value of 0.91, which falls within the “marvelous” range, thereby providing strong evidence for the appropriateness of factor extraction. Inspection of individual KMO values revealed that all items exceeded 0.86, further reinforcing the adequacy of the dataset for factor analytic techniques.

**Figure 1:**
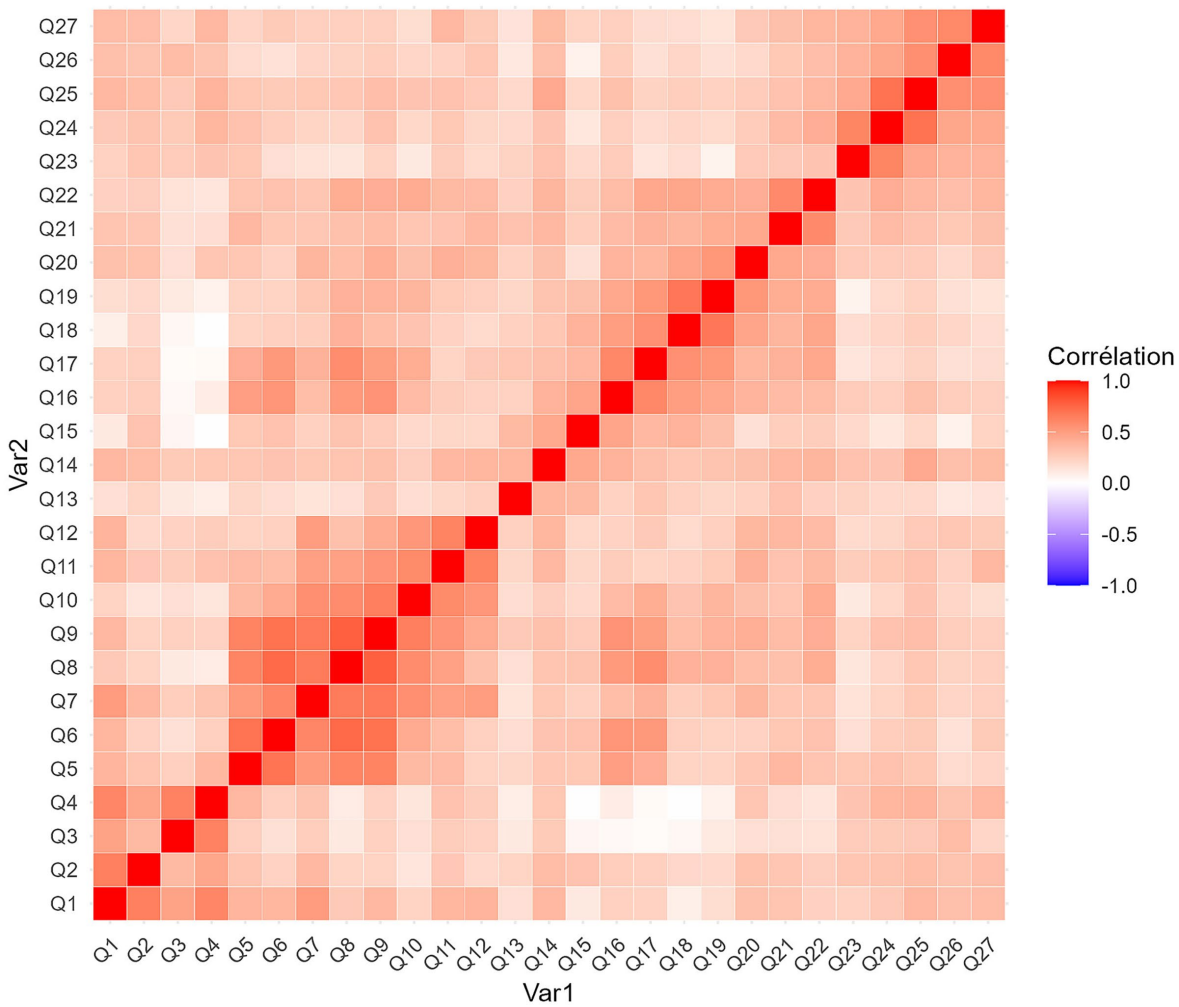
Polychoric correlation matrix of QPE4PL*impact* items.

As shown in Figure 1, strong correlations were observed among items within the same factor, particularly between consecutive items, whereas negligible associations were found between items belonging to unrelated factors (e.g., item 17 and item 23). This pattern is consistent with the hypothesized multidimensional structure of the QPE4PL*impact* questionnaire.

**Figure 2:**
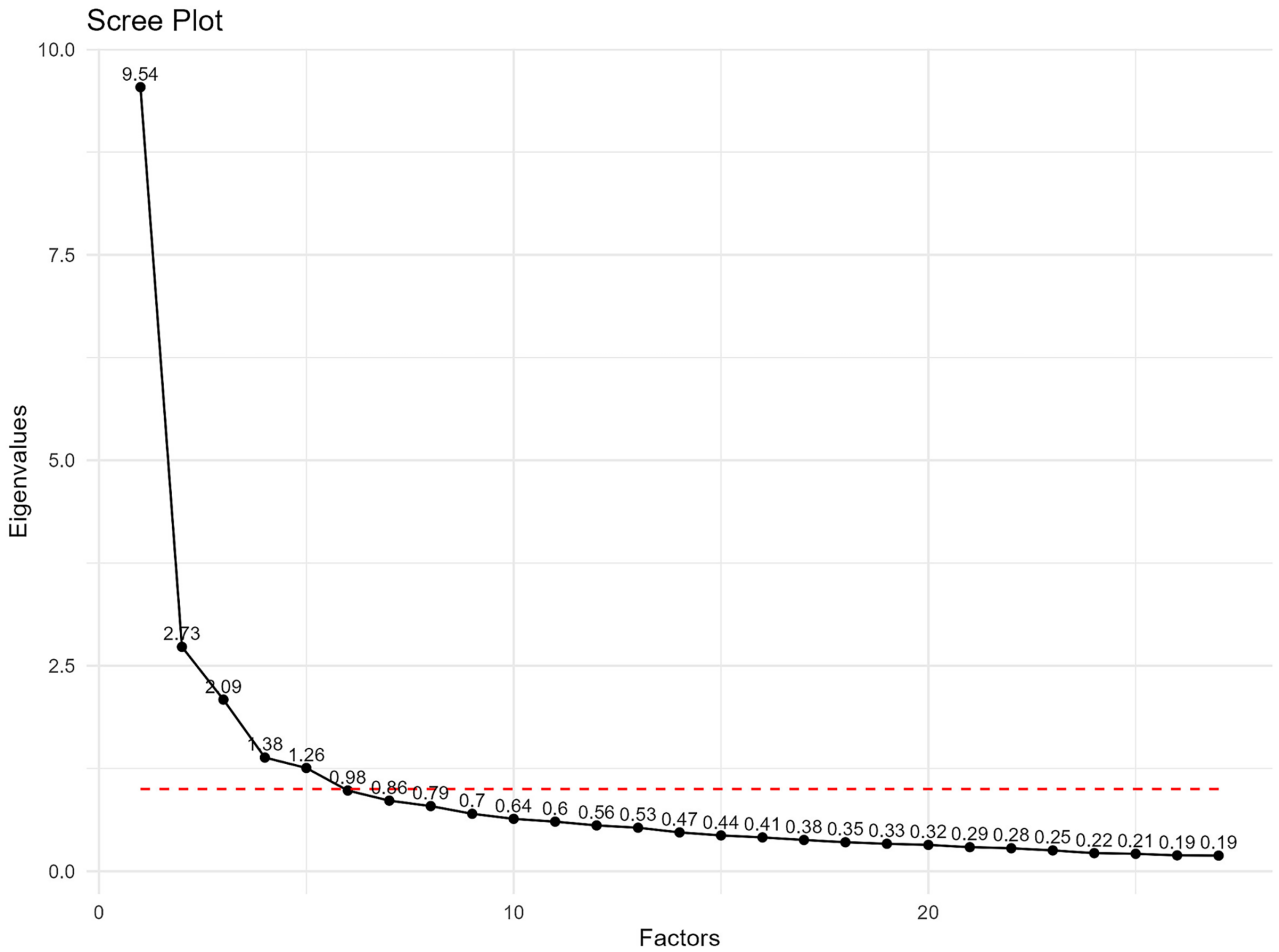
Eigenvalue scree plot supporting a four-factor solution.

Initial inspection of the scree plot suggested the presence of five latent factors. However, results from the preliminary EFA indicated that a more parsimonious four-factor solution yielded a clearer structure and stronger theoretical interpretability (Figure 2).

### Exploratory factor analysis

The EFA, conducted on the initial 27 items of the QPE4PL*impact* scale, revealed the necessity for item reduction to improve model fit and conceptual coherence. Three rounds of EFA were conducted. After the first round, five items were removed: items 7, 16, and 17 exhibited cross-loadings on multiple factors, whereas items 13 and 15 did not reach sufficient factor loadings. Following the second round, items 11, 12, and 14 were excluded for similar reasons. The third round of EFA resulted in a robust four-factor structure, comprising a minimum of four items per factor and a total of 19 items in the finalized questionnaire.

Table 2 displays the EFA results (direct oblimin rotation, *n* = 328), showing factor loadings for the 19 retained items across the four PL domains.

**Table 2:** Results of the exploratory factor analysis via direct oblimin rotation (*n* = 328).

Items	1	2	3	4
1. …je pense que je suis efficace dans cette activité. [I think I am effective in this activity.]				0.79
2. …je réalise mieux qu’avant certaines actions efficaces dans l’activité. [I perform certain effective actions in this activity better than before.]				0.59
3. …j’ai pu réaliser l’ensemble des exercices sans m’arrêter. [I was able to complete all the exercises without stopping.]				0.61
4. …je réalise la majorité des exercices demandés sans difficulté. [I carry out most of the required exercises without difficulty.]				0.80
5. …j’ai envie de recommencer cette activité la semaine prochaine pour continuer de progresser. [I want to do this activity again next week to keep improving.]	0.68			
6. …j’ai envie de pratiquer cette activité volontairement ailleurs qu’en cours d’EPS. [I want to voluntarily practice this activity outside of PE lessons.]	0.89			
8. …je pense que je pourrais me sentir mieux si je continuais cette activité. [I think I could feel better if I continued this activity.]	0.88			
9. …je ressens un bien-être personnel à la suite de ce cours. [I feel personal well-being after this lesson.]	0.82			
10. …j’ai appris à mieux gérer mes émotions pendant ce cours. [I learned to better manage my emotions during this lesson.]	0.52			
18. …j’ai eu l’impression de faire partie d’un groupe. [I felt like I was part of a group.]			0.73	
19. …j’ai appris à mieux communiquer avec mes camarades. [I learned to communicate better with my classmates.]			0.85	
20. …j’ai l’impression d’avoir appris à agir de manière autonome. [I feel like I have learned to act independently.]			0.58	
21. …j’ai conscience que mon activité peut influencer celle des autres. [I am aware that my activity can influence that of others.]			0.44	
22. …je trouve que j’ai été amené à prendre des decisions et à assumer des responsabilités. [I feel that I was led to make decisions and take on responsibilities.]			0.44	
23. …j’ai bien compris les règles de l’activité. [I clearly understood the rules of the activity.]		0.63		
24. …je connais les bonnes actions pour réussir dans cette activité. [I know the right actions to succeed in this activity.]		0.82		
25. …je sais comment éviter les erreurs dans cette activité. [I know how to avoid mistakes in this activity.]		0.77		
26. …je peux identifier mon niveau. [I can identify my level.]		0.66		
27. …je suis capable de me fixer un objectif accessible. [I am able to set myself an achievable goal.]		0.63		

Following the EFA, the emerging statistical structure demonstrated strong convergence with the theoretical framework established during the dimension identification and item generation phases. Specifically, the EFA confirmed the presence of four distinct latent factors, each grouping items in a manner consistent with their hypothesised dimensional affiliation. After removal of cross-loading items and those with insufficient communalities, items Q1–Q4 clustered on the first factor, corresponding to the physical domain; items Q5, Q6, Q8, Q9, and Q10 loaded strongly on the second factor, aligned with the psychological-affective domain; items Q18–Q22 formed a coherent third factor reflecting the social domain; and items Q23–Q27 loaded together on the fourth factor, representing the cognitive domain.

Table 3 presents a comprehensive mapping of the PL domains and subdomains retained from the Delphi consensus (Gandrieau et al., 2023), alongside the corresponding questionnaire items that were either retained or excluded during the entire development process, including item generation, cognitive interviews, and factor analyses. This synthesis clarifies the theoretical and empirical decisions underlying the final QPE4PL*impact* questionnaire.

**Table 3:** Mapping of PL domains, subdomains, and questionnaire items retained or rejected during scale development.

Domain	Subdomain	Item generation	Post-EFA status
Physical	Movement competencies	Q1	Retained
	Movement competencies	Q2	Retained
	Movement competencies	Q3	Retained
	Movement competencies	Q4	Retained
	Objective tests	Discarded	
	Perceived Aquatic competencies	Discarded	
	Bicycle competencies	Discarded	
	Motor creativity	Discarded	
Psychological-affective	Motivation	Q5	Retained
	Motivation	Q6	Retained
	Self-Esteem	Q7	Rejected
	Self-Esteem	Q8	Retained
	Enjoyment	Q9	Retained
	Emotional competence	Q10	Retained
	Belief	Q11	Rejected
	Confidence	Q12	Rejected
Social		Q13	Rejected
		Q14	Rejected
		Q15	Rejected
		Q16	Rejected
		Q17	Rejected
	Social acceptance	Q18	Retained
	Relational skills	Q19	Retained
	Self-management	Q20	Retained
	Social awareness	Q21	Retained
	Responsible decision-making	Q22	Retained
Cognitive		Q23	Retained
		Q24	Retained
		Q25	Retained
		Q26	Retained
		Q27	Retained

### Confirmatory factor analysis

The four-factor structure of the QPE4PL*impact* identified through EFA was subsequently tested on the independent confirmatory subsample (*n* = 165) via CFA. The model was estimated using the adjusted WLSMV estimator, implemented in the *lavaan* package in R.

Fit indices indicated a good correspondence between the hypothesized model and the observed data. The scaled Comparative Fit Index (CFI = 0.978) and scaled Tucker-Lewis Index (TLI = 0.974) both exceeded the pre-established acceptability threshold (> 0.85). The Root Mean Square Error of Approximation (RMSEA = 0.073) fell below the 0.08 criterion, and the Standardized Root Mean Square Residual (SRMR = 0.082) was also within the acceptable range. Together, these values provide strong cross-validation evidence for the stability and adequacy of the four-factor solution.

### Second-order confirmatory factor analysis

**Figure 3:**
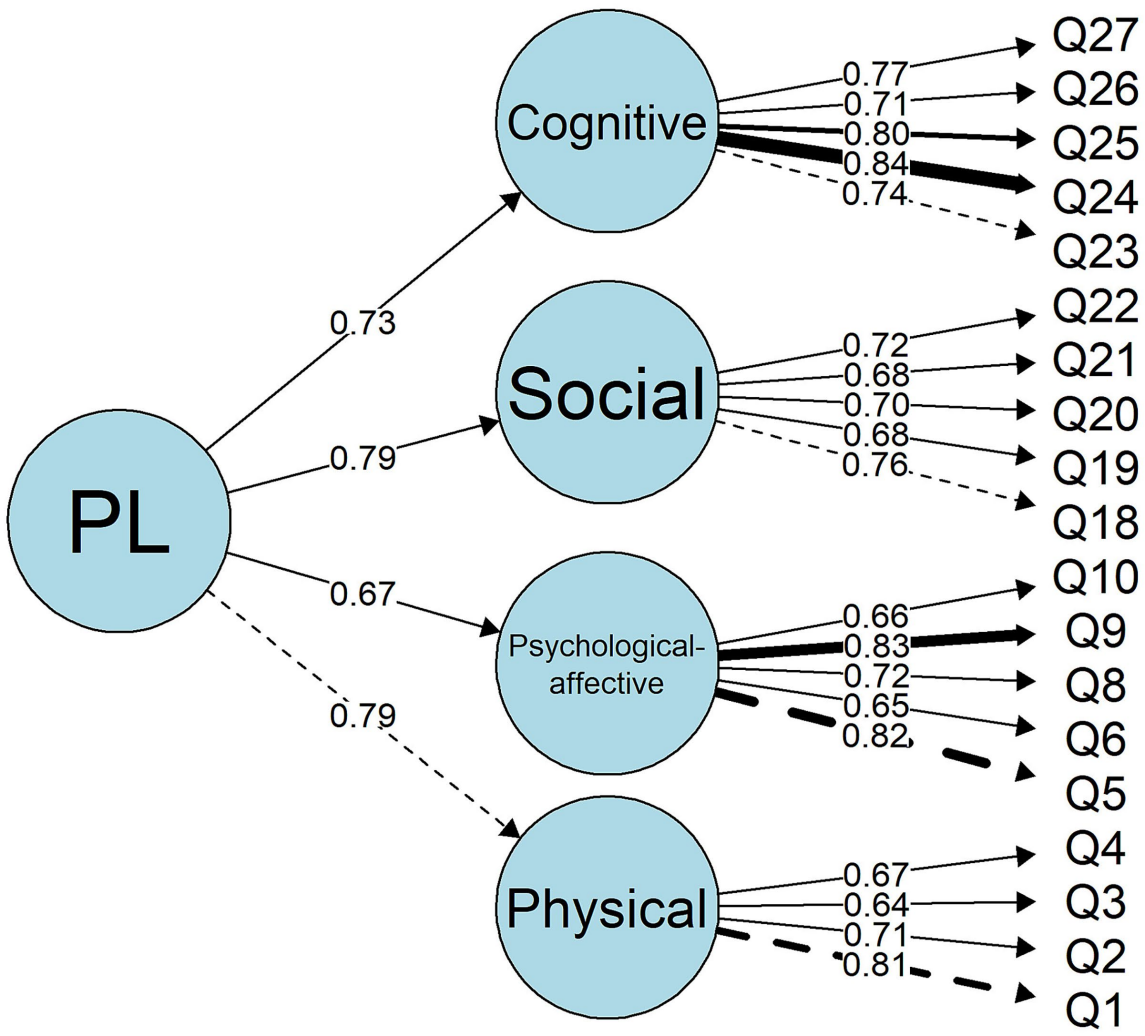
Hierarchical CFA model of the QPE4PL*impact*, showing the four first-order domains (physical, psychological-affective, social, cognitive) loading onto the higher-order construct of PL.

A second-order factor model was then tested to examine the presence of an overarching PL construct. In this hierarchical model, the four first-order factors loaded significantly onto a single higher-order factor. Model fit remained robust: CFI = 0.969, TLI = 0.964, RMSEA = 0.087, and SRMR = 0.091. While the SRMR slightly exceeded the predefined criterion, the remaining fit indices supported the adequacy of the hierarchical structure. The hierarchical factor structure of the QPE4PL*impact*, comprising both the four first-order domains of PL and the overarching second-order construct (PL), is illustrated in Figure 3.

### Internal consistency

Internal consistency was assessed using Cronbach’s alpha coefficients. The QPE4PL*impact* demonstrated acceptable to good internal consistency across all domains, with *α* values ranging from 0.753 to 0.833. Specifically, Cronbach’s alpha coefficients were 0.753 for the physical domain, 0.822 for the psychological-affective domain, 0.767 for the social domain, and 0.833 for the cognitive domain. The overall scale demonstrated good internal consistency (α = 0.885).

## Discussion

This study aimed to develop and validate the QPE4PL*impact* questionnaire, a situational instrument designed to assess the immediate impact of PE lessons on high school students’ PL. The findings provide strong support for the psychometric robustness of the instrument. The EFA revealed a clear four-factor structure corresponding to the physical, psychological-affective, social, and cognitive domains of PL, while the CFA confirmed the stability of this structure on an independent sample. Furthermore, the second-order CFA supported the presence of a higher-order latent construct, indicating that these domains coherently integrate into a unified situational PL construct. This hierarchical structure provides empirical support for the multidimensional yet integrated nature of PL, which is frequently emphasized in theoretical literature.

The presence of distinct but interrelated domains reflects the conceptualization of PL as a holistic construct encompassing physical, psychological-affective, cognitive, and social components, while the higher-order factor suggests that these domains collectively contribute to a broader experiential understanding of movement engagement. In this sense, the psychometric results obtained in this study reinforce the theoretical proposition that PL is both multidimensional and unified, rather than a simple aggregation of independent abilities.

### Theoretical interpretation of situational physical literacy assessment

A defining strength of this study lies in its explicit theoretical anchoring within the existentialist and phenomenological foundations of PL, an approach rarely emphasized in validation research. By grounding the development of the QPE4PL*impact* questionnaire in these perspectives, this work responds to repeated calls for measurement approaches that are both philosophically coherent and empirically robust (Edwards et al., 2018; Merleau-Ponty, 1945; van Manen, 1990; Whitehead, 2001). While many previous instruments have prioritized pragmatic psychometric considerations, few have foregrounded the lived, subjective experience of movement that lies at the heart of PL scholarship. In this regard, the present study contributes to bridging the gap between philosophical foundations and empirical measurement.

This contribution must, however, be understood in light of an inherent tension within the field between quantitative approaches and the fundamentally experiential, subjective nature of PL. Scholars have warned that traditional quantitative measures may fragment the holistic and dynamic nature of PL into static variables, potentially misaligning with the phenomenological emphasis on personal meaning and situated experience. Despite the robust statistical indices obtained in this study **—**such as factorial validity and internal consistency**—**the reliance on self-reported data collected immediately after a PE lesson raises important questions regarding what is actually being measured. Specifically, it remains necessary to consider whether such situational assessments capture PL as conceptualized in the literature**—**a lifelong and evolving journey**—**or whether they primarily reflect an immediate state of engagement, satisfaction, or situational interest triggered by a specific learning environment (Chen, 1996; Green et al., 2018; Zhu et al., 2009). From this perspective, the QPE4PL*impact* occupies a paradoxical position: it seeks to quantify the impact of a single pedagogical episode within a construct defined by its continuity and development over time (Carl et al., 2022; Edwards et al., 2018).

Clarifying the meaning of the data produced by this tool is therefore essential. In line with literature on situational interest, post-lesson measures may be highly sensitive to immediate contextual factors, including task design, teacher behaviors, and group dynamics (Zhu et al., 2009). Although the questionnaire items were carefully designed to access students’ lived experiences in a holistic manner, responses may still be influenced by factors such as novelty, enjoyment, or activity type rather than reflecting deeper changes in PL. This raises an important issue of ecological validity: can self-reported data collected immediately after a single lesson adequately capture the richness of lived experience emphasized in phenomenological theory, or does it risk conflating momentary engagement with more enduring developmental processes (Edwards et al., 2018)? Existing literature suggests that situational questionnaires can provide valuable insights for immediate pedagogical reflection, but they should not be interpreted as direct indicators of long-term PL development (Badiola-Lekue et al., 2025).

### Contributions and practical implications of the QPE4PL*impact*

Despite these conceptual challenges, the present study offers several important contributions. First, the development process followed a transparent and rigorous methodological approach. The questionnaire was grounded in an international Delphi consensus, refined through cognitive interviews with students, and validated using robust psychometric procedures including EFA, CFA, and second-order factor modeling. This methodological transparency clarifies the conceptual boundaries of the instrument while explicitly acknowledging both its strengths and its limitations.

Second, the QPE4PL*impact* provides a practical and innovative tool for multiple stakeholders in the field of PE. For teachers, the instrument offers rapid feedback on the perceived impact of pedagogical choices within a specific lesson. Such information may support reflective teaching practices and inform adjustments to instructional design in order to better support students’ engagement and development. For researchers, the tool offers a complementary approach to existing global PL assessments by capturing the immediate, context-dependent dimensions of students’ experiences during PE. For policy-makers and educational institutions, its ease of administration-requiring less than 10 min and minimal equipment-facilitates its integration into routine evaluation processes aimed at improving the quality of PE programs.

Beyond its immediate practical applications, the QPE4PL*impact* also contributes to addressing a broader theoretical paradox within PL research. Both the conceptualization of PL as a lifelong developmental journey and Bronfenbrenner and Morris’s bioecological model (Bronfenbrenner and Morris, 1998, 2006) emphasize long-term processes shaped by repeated interactions between individuals and their environments. At first glance, the situational nature of the QPE4PL*impact* may appear inconsistent with such long-term perspectives. However, from an existentialist standpoint, individual experiences play a decisive role in shaping meaning and development. In this sense, each measurement produced by the questionnaire can be interpreted as a situational snapshot reflecting a specific moment within the evolving relationship between the learner and their context.

When considered within a broader research design, this situational perspective may become a valuable opportunity rather than a limitation. Interest development models (e.g., Hidi and Renninger, 2006) illustrate how positive situational experiences can gradually contribute to the formation of enduring personal interests. Similarly, repeated administration of the QPE4PL*impact* across multiple lessons or learning environments may allow researchers and practitioners to map the quality of students’ experiences over time and examine how stimulating pedagogical contexts accumulate to support the development of a more integrated and global form of PL. In this way, the instrument may serve not only as a momentary indicator of lesson impact but also as a tool for investigating the pedagogical conditions that facilitate the transition from situational experiences toward lifelong PL development.

### Limitations and future research directions

Several limitations should nevertheless be acknowledged. Alongside limitations already discussed —including potential social desirability bias, the influence of emotional states, contextual specificity, and the early stage of field validation— three additional aspects deserve particular attention.

First, the nature of the physical activity itself may influence students’ responses. Different activities vary in competitiveness, novelty, and perceived difficulty, which may strongly shape students’ perceptions of their experience during a given lesson. This activity context bias makes it difficult to fully disentangle the influence of instructional design from the characteristics of the activity itself (Zhu et al., 2009). Future research should therefore consider repeated measurements across diverse types of physical activities in order to establish more stable situational profiles.

Second, the questionnaire was developed and validated within the context of compulsory school PE. Its ability to capture situational expressions of PL in extracurricular or community-based physical activity settings remains untested. This limitation restricts the external validity of the instrument with regard to adolescents’ broader physical activity experiences beyond the school environment (Edwards et al., 2018).

Third, the EFA process led to the removal of several items representing domains previously identified as relevant during the Delphi study conducted by Gandrieau et al. (2023). Although this reduction improved statistical coherence and model fit, it may limit the ability of the final questionnaire to fully represent all theoretically relevant subdomains of PL identified by field experts.

Future research should therefore pursue several complementary directions. Longitudinal studies could investigate how situational responses measured by the QPE4PL*impact* relate to longer-term trajectories of PL development. Integrating qualitative methods-such as interviews or focus groups-could also provide deeper insight into the meanings underlying students’ responses and enrich the interpretation of quantitative scores. Furthermore, triangulation designs comparing student perceptions with teacher observations or external evaluations of lesson quality may help clarify the pedagogical processes underlying situational PL experiences.

In addition, the questionnaire shows strong potential for international adaptation. Although the present study was conducted exclusively in France, the conceptual foundations of the QPE4PL*impact* are aligned with widely recognized PL frameworks and theoretical models. Future research could therefore focus on cross-cultural validation processes involving linguistic translation, cultural adaptation, and psychometric testing in diverse educational contexts. Such efforts would contribute to the broader international dialogue on situational approaches to PL assessment.

## Conclusion

In conclusion, the present study provides initial evidence supporting the validity of the QPE4PL*impact* as a situational measure of PL in PE contexts. Through a rigorous development process combining theoretical grounding, cognitive interviews, and psychometric validation, the instrument demonstrated a coherent four-domain structure encompassing physical, psychological-affective, social, and cognitive aspects of PL. By focusing on students’ immediate experiences within PE lessons, the QPE4PL*impact* offers researchers and practitioners a novel tool for assessing the situational impact of PE on PL development. Future research should continue to examine its reliability, sensitivity to change, and applicability across diverse educational contexts.

Future research should investigate whether situational PL scores are influenced by the characteristics of different PE activities and assess measurement invariance across activity contexts to strengthen the interpretability and comparability of scores.

## Data Availability

The datasets presented in this study can be found in online repositories. The names of the repository/repositories and accession number(s) can be found at: https://doi.org/10.5281/zenodo.19221973.
